# Finding Dutch natives in online forums

**DOI:** 10.1080/20961790.2018.1482042

**Published:** 2018-09-28

**Authors:** Bernard van den Boom, Cor J. Veenman

**Affiliations:** a Leiden University, Leiden Institute of Advanced Computer Science (LIACS), Leiden, The Netherlands;; b Netherlands Forensic Institute, The Hague, The Netherlands;; c TNO, The Hague, The Netherlands

**Keywords:** Forensic data science, text mining, author profiling, corpus creation, big data, open source intelligence, native language verification

## Abstract

Law enforcement agencies have a restricted area in which their powers apply, which is called their jurisdiction. These restrictions also apply to the Internet. However, on the Internet, the physical borders of the jurisdiction, typically country borders, are hard to discover. In our case, it is hard to establish whether someone involved in criminal online behavior is indeed a Dutch citizen. We propose a way to overcome the arduous task of manually investigating whether a user on an Internet forum is Dutch or not. More precisely, we aim to detect that a given English text is written by a Dutch native author. To develop a detector, we follow a machine learning approach. Therefore, we need to prepare a specific training corpus. To obtain a corpus that is representative for online forums, we collected a large amount of English forum posts from Dutch and non-Dutch authors on Reddit. To learn a detection model, we used a bag-of-words representation to capture potential misspellings, grammatical errors or unusual turns of phrases that are characteristic of the mother tongue of the authors. For this learning task, we compare the linear support vector machine and regularized logistic regression using the appropriate performance metrics *f*_1_ score, precision, and average precision. Our results show logistic regression with frequency-based feature selection performs best at predicting Dutch natives. Further study should be directed to the general applicability of the results that is to find out if the developed models are applicable to other forums with comparable high performance.

## Introduction

The police and intelligence agencies undoubtedly struggle with the massive amount of textual content that is posted online, some of which has a criminal nature. Searching for this type of postings on the whole web is a daunting task. It is clear that criminals use the Internet as a medium to sell illegal arms or drugs as well as more extreme cases such as offering hitmen services. We are especially interested in content posted on the so-called dark web, which is more often criminal in nature. Most commonly found crime related content includes black markets, child pornography, fraud, or mail order services.

Apart from the enormity of the internet, there is another problem for the agencies. The Dutch law enforcement agencies cannot follow up on users involved in criminal online activities that are outside their jurisdiction. Indeed, finding Dutch citizens could be as easy as language detection on the online postings [1]. However, the Internet is a global meeting place, where many of the relevant public forums contain mostly English posts. In other words, intelligence agencies deal with the magnitude of the Internet featuring criminal content from users with a wide variety of nationalities which is only partially relevant to them. A system to support the identification of Dutch citizens among web users is urgently needed.

In case the traces of the users are all *English* posts, the problem of finding Dutch citizens boils down to identifying English posts written by Dutch natives. The problem is closely related to the Native Language Identification (NLI) problem [2]. In NLI, the problem is to determine the native language of an author, where the native language can be any of a given set of languages. The idea is that texts written by non-native speakers include hidden clues that betray their native language. Writers are prone to misspellings, grammatical errors, or unusual turns of phrases that are characteristic of their mother tongue. In this study, the first goal is to leverage the power of machine learning and automated text analysis to uncover these clues in order to detect English texts written by Dutch authors. From the standpoint of a law enforcement agency, the problem here is to determine if a poster is in their jurisdiction yes or no. In other words, we are dealing with a *Native Language Verification* (NLV) problem.

Like others with related problems, we follow the machine learning paradigm to develop a method to differentiate Dutch natives’ posts from non-Dutch natives’ posts. For such a data-driven approach, we need to have a representative text corpus with ground truth of the native language.

From a machine learning perspective, the NLV problem can be posed as a two-class classification problem, where the classes are 1 and 0 with respect to being a Dutch native. In contrast, the general NLI problem results in a multi-class classification problem, where every possible native language forms a class. Two-class classification problems have a number of advantages that we exploit in this study. First, several top performing classifiers for high-dimensional text analytics problems are principally designed for two-class problems, such as the Support Vector Machine (SVM) and logistic regression. Applying such methods to multi-class problems requires adaptation with a multi-class wrapper at the cost of optimality. Second, for the NLI task, typically accuracy is used as performance measure [3]. This measure is prone to class-imbalance [4], which is typically present in this domain. For two-class problems, tried and tested measures exist that are robust against class-imbalance and allow for easy interpretation, such as the ROC curve [5] and its aggregated Area Under the ROC Curve (AUC), and precision and recall [6]. Moreover, these metrics are well-suited for measuring the performance to rank the posts from most probable native Dutch to least probable Dutch.

This research has two main contributions:

1. We propose a recipe for the construction of a corpus with English posts with ground truth labels for native Dutch and for native non-Dutch. Clearly, this can be translated to any other language.
2. We propose a method for ranking posts, or the users thereof, for the Native Language Verification problem.

This paper has the following structure. The next section poses the problem we deal with formally. Then the *Related Work* section discusses the literature related to our work. Section *Corpus* describes the corpus collection process. Section *Method* gives an overview of the machine learning related method, including feature extraction and model learning. In the following sections, we report the evaluation method and results of the experiments we did. We conclude with a discussion, conclusions, and an outlook.

## Problem statement

The problem we deal with in this study is to provide law enforcement agencies a tool to distinguish written online English text by Dutch authors from English text written by non-Dutch authors. More precisely, for a collection of English posts by different authors, the task is to rank them from most probable native Dutch to least probable native Dutch author. For this task, we only exploit the uttered written text, because possible available metadata, such as IP addresses, can be hard to follow-up for various reasons, which is outside the scope of this research.

## Related work

The native language recognition problem touches upon various text classification tasks, such as author profiling [7], authorship attribution [8], programmer identification [9], review classification [10], and Twitter sentiment analysis [11].

Of the contemporary classification problems, native language identification comes clearly closest to our problem, see for example [2,12,13]. Much of native language identification research use *learner English documents* as training corpus [2,3,14]. A popular such resource is a Test of English as a Foreign Language (TOEFL) corpus, which contains texts that are used to test students’ readiness to study in an English-speaking country [15].

Most of these document classification tasks proceed in a fairly standard way [16]. First, they collect the textual data. Then, in a preprocessing process, the texts are tokenized, stop-words are removed and words are stemmed. What often follows is an indexation step, for example by using the vector space model, creating vectors of characters, words, combinations of characters, or combinations of words. The resulting feature space can become really huge. The resulting dimensionality problem, or curse of dimensionality, is commonly addressed by using feature selection techniques based on information gain, term frequency, or performing the chi-square test [17–19].

After the data are prepared in some feature vector format, a machine learner can be applied to obtain a text classifier. Many different models have been used in the past, some with more success than others. Often regularized machine learners are used (in addition to feature selection schemes) to deal with the dimensionality of the problem, such as the SVM [20], Lasso [21], and regularized logistic regression [22]. Other used methods are naive Bayes [11,23], the *k*-nearest-neighbor classifier [24], decision trees [11], and random forest classifiers [25].

## Corpus

In this section, we discuss our corpus collection recipe. To obtain a representative corpus of sufficient size for the NLV task in online forums with potential criminal activities, we would preferably use content from the dark web. However, obtaining this data is hard and sometimes even illegal. Alternatively, the Reddit platform [26] is a suitable alternative that allows for easy access of their content. Below we elaborate on how we gathered the data and obtained native Dutch and native non-Dutch labels for the users of the platform.

### Reddit

Reddit is an online community where registered users can, among other things, submit content, vote on submissions, and comment on them. There are currently around 40 million user accounts. Visitors can also view most content without having a user account [26]. Almost all parts of Reddit are in English; the majority of the users on Reddit are from the U.S. Exact numbers on where Reddit users come from are not publicly available. In accordance with the Problem Statement, in this work, we do not use meta-data, which is hard to obtain anyway with Reddit.

### Data acquisition

Reddit provides an Application Programming Interface (API) [27] of which the code is open source. It supports many methods including gathering data from particular subreddits and users by making a variety of calls. A subreddit is a sub forum on Reddit, which collectively form Reddit. For this research, comments are acquired through these methods and by a Python package that allows for simple access to Reddit’s API [28]. We create a labeled dataset where all comments from a user are collected into one document. Law enforcement agencies need to know if a user is within their jurisdiction, so they do not need to classify individual comments. In authorship identification literature, this is called profile-based classification, unlike its opposite instance-based classification [29], where we would consider each comment from a user as a separate instance. The advantage is that larger chunks of text can be used for the task.

#### Dutch users

In order to correctly classify users as either Dutch or non-Dutch, we first obtain data of which it can be known with reasonable certainty that they are native Dutch users. The data are gathered by the following process:

1. Because the vast majority of subreddits is non-Dutch, a list of the largest Dutch subreddits that could be found were gathered manually. We end up with around 900 users from some of the largest subreddits that have been observed to be in Dutch. We make the assumption here that users that post comments in Dutch are Dutch.
2. For each of these Dutch users, we extract the *comment* the *user* has commented in a particular *language* as detected by Googles language-detection ported to Python [30] in a certain *subreddit*. We ended up with roughly 540 000 records from Dutch users.
3. As a last step, we remove the records that include comments in Dutch or any non-English language that the language detector was able to come up with. The result is around 400 000 comments in English from assumed Dutch users.

Although every comment is passed through the language detector, it does not seem to be fully effective. The language detector wrongly identifies some comments – and thus some users – as speaking a certain language. It does so because (1) some comments are too short to classify and (2) some comments include uncommon characters such as emoticons or Reddit specific slang. How much errors the language detector actually makes is hard to say because the number of comments is too high to check manually. It might label some short comments correctly, others incorrectly. Indeed, the language detector recommends using texts of more than 10–20 words [30].

#### Non-Dutch users

Because we only distinguish between Dutch natives and non-Dutch natives we assume that all users that do not post comments in Dutch are non-Dutch users. Indeed, those Dutch users who only post in English will wrongly be considered English. However, those users will strongly be outnumbered by truly non-Dutch, so most likely the learning algorithms will not be hampered by this [31]. The exact process of gathering non-Dutch user data and the attempt at detecting their native language is as follows:

1. In order to come up with an initial list of non-Dutch users, we take the top 200 of subreddits, where Dutch users from the Dutch user data have posted in (in English). This allows for comments not dealing with widely varying themes, that would likely lead to overfitting the data later on. The idea here is that the dataset becomes more homogenous if we gather comments from subreddits where both Dutch natives and non-Dutch users comment in. From each of these subreddits in the created subreddit list, we gather as much users as possible, and filter out all the Dutch users we have found in our Dutch user data. We now have a temporary list of 90 000 non-Dutch users. That is 100 non-Dutch users for every 1 Dutch user.
2. Step 2 is identical to Step 2 of the Dutch users data gathering process, except we gather less comments per user, because there are simply more users to gather data from. The gathering of this data takes a couple of days and results in a large csv file of around 2.5 GB with the same header fields as the Dutch user data set.

Now, as the language detector is proven to be not as effective on short comments and or “weird” characters and there is no way of checking how good it works, an attempt is made to classify subreddits, instead of single comments, in a certain language. If we assume a user is labeled a language *l* if the subreddit is in a foreign language and some user posts in that subreddit, we can eventually match users to a language. We hope that subreddits contain enough comments to correctly find out its language and thus the user’s language. Often, subreddits are in one language or another, not mixing languages in the particular subreddit. Subreddit language is determined as follows:

1. Create a list of unique subreddits from the non-Dutch user data. These are all the subreddits the presumably non-Dutch users comment in.
2. Of each subreddit, 30 comments are grabbed and concatenated. 30 proves to yield a set of comments large enough to be tested by the language detector.
3. On that concatenation we apply the language detector.
4. The language that is detected is paired with the unique subreddit. The result is a dataset with subreddits and their corresponding language.

After we have determined the subreddit language of all the subreddits that our assumed non-Dutch users posted in, we remove all users that post in Dutch subreddits from our non-Dutch data set and add them to our Dutch data set.

Finally, we concatenate all comments belonging to a user together into a single text fragment. As a consequence of concatenating comments, it will most likely also be easier for the machine learning algorithm to eventually classify documents. That is, they contain more data to extract information from. This is also why users with concatenated texts shorter than 2 000 characters are removed. This reduces potential detection errors on short texts that only include illegible symbols.

After closer examination by hand, we notice that there remain quite some comment sets that include sentences in Dutch. The language detector apparently has not been able to detect these comments individually. For that reason we run the language detector again, now on the comment set, removing another 100 or so instances of Dutch users. We attempt to delete as much Dutch as possible from the individual comments during gathering first and from the comment sets later.

To summarize, we have gathered data in two main steps. First, we have largely by hand created a list of Dutch users (a minority on Reddit) that post in Dutch subreddits. We then composed a list of preliminary non-Dutch users from the subreddits Dutch users post in. We double checked whether they were non-Dutch by matching the users with the Dutch data, and by language detecting the subreddits they post in. Lastly, we removed rows of comment sets that were put through the language detector once again and were flagged non-English. The data are now ready to be transformed into features, and is in the format:

[user] [aggregated comments] [nl/other]

The user is the name of the user, the aggregated comments are all the comments gathered from a particular user and concatenated together and the last column is one of the two languages *Dutch* and *English* (which in fact is everything non-Dutch).

## Method

### Feature extraction

In order to be able to use many of the machine learning algorithms, text data need to be converted into numerical feature vectors. The bag-of-words model is an approach widely used in the field of natural language processing [32–34]. With such an approach we essentially disregard word order: every comment is taken as a multiset of its words, keeping only track of the frequency of each word. This frequency is then used as a feature for training the classifier.

Our toolkit provides many parameters that can control how to preprocess data, besides transforming the data with term frequency times inverse document frequency (tf-idf). Parameters include stripping accents, determining whether to remove stop words, lowercasing all characters and setting an *n*-gram range and whether to use character *n*-grams or word *n*-grams. Much of the parameters have been kept as simple as possible and as close to contemporary research as possible. We elaborate on our steps and choices below.

#### Tf-idf

First, we tokenize each comment collection into words and then use a technique called tf-idf. With this technique, we quantify the importance of words in documents (comment collections) by calculating weights for them. The most frequent words are not always the words bearing the most information. Indeed, they are often words that are so called stop words (“the”, “and”, “or” etc.). Often, rarer words can give more information whether or not some user is Dutch or not. For example, Dutch users might make certain spelling mistakes that non-Dutch users almost never make.

The calculation of tf-idf is as follows:

(1) $$TF_{ij}=\frac{f_{ij}}{\max_{k}f_{kj}}.$$

with *f_ij_* the frequency of word *i* in document (concatenated comments) *j*. So, the term frequency of term *i* in the document is *f_ij_*, normalized by dividing it by the frequency of the most frequent word in the document. The most frequent term in comments *j* gets a term frequency of 1. The inverse document frequency $IDF_{i}=\;{\text{log}}_{2}(\frac{N}{n_{i}})$ with *N* the total number of document and *n_i_* the number of documents containing word *i*. Tf-idf then becomes $TF_{ij}\times IDF_{i}$. Higher values for tf-idf are obtained by a high frequency for the document, but low frequency over all documents [35].

#### *N*-grams

The use of *n*-grams is common in text classification tasks, see for example [36,12]. With word *n*-grams we extract *n* contiguous words from a document. Similarly, we could use syllable or character *n*-grams. *n*-grams are aimed at eventually providing us with a prediction of the next word or character in a sequence of words (for example the next word in a sentence). Although fairly simple, *n*-grams have proven to be very effective in many applications. It was not feasible to test a wide variety of *n*-gram ranges during our research, although that might have improved our final results. Initial experiments showed that word *n*-grams performed better than character *n*-grams. Therefore, we continued the research using word *n*-grams. Furthermore, we decided to use word unigrams, i.e. word *n*-grams of size 1, because using bigrams or even trigrams lead to implementation problems caused by the high number of resulting features.

#### Other parameters

We have decided to lowercase all characters. We lowercase to prevent our vocabulary from increasing significantly in size without showing an increase in performance according to early tests. We do not strip any accents or remove any stop words. Stop words are usually removed in text classification, but in our case it might be useful, namely Dutch users might choose different stop words than non-Dutch users.

Lastly, we binarize the term frequency in tf-idf. Purely by some initial experiments, this parameter setting has proven to be giving better performance. Term frequencies are also by default normalized by using the *l*2-norm.

After feature extraction we end up with as many as 2.3 million features.

### High dimensionality

About 2.3 million features can lead to some problems. First, storing so many features requires a vast amount of memory storage. Second, high dimensionality might lead to worse performance or even overfitting, when dimensionality increases while training samples remain fixed [37,38]. Overfitting might be a direct result of this so called curse of dimensionality. Fortunately both our models are linear and regularized which – assuming properly tuned penalty parameters – makes them more resistant to overfitting [39]. We further cover two ways of dealing with the high dimensionality of our data.

One common way of addressing high dimensionality is feature selection. Feature selection has in many cases proven to be useful to simplify the eventual model without giving up performance and even improve generalization accuracy and avoid “overfitting”. In the recent literature, mutual information, information gain, term frequency, and Chi-squared are among the feature selection techniques that have proven most effective [40]. We experiment with chi-squared and term frequency. For the term frequency, we limit the maximum number of features by ordering by term frequency in our corpus. With chi-squared feature selection the statistical chi-squared test is used to select features [17].

Regularization is another way of dealing with the curse of dimensionality. For instance, feature selection can be done by *L*1 regularization. We, however, use *L*2 regularization, because *L*1 regularization gives worse results. Regularization ensures the weights the model gives are not fit too well: weight values are penalized by regularization. Simply put, the difference between the two types of regularizations is that *L*1 regularization can shrink weights to zero, effectively eliminating them, while *L*2 regularization shrinks the weights too but eliminates none [21].

### Model learning

As has been mentioned in section *Related work*, machine learning algorithms that are often used with text data include naive Bayes, or other Bayesian models, SVMs, logistic regression and the *k*-nearest-neighbor classifier. The latter algorithm generally is fairly slow and memory demanding compared to the others. For that reason, it has not been included in the experiments. The Naive Bayes classifier showed limited performance on this dataset in initial experiments, so we also left it out. Finally, the two remaining methods were tested, being regularized logistic regression (LR) and the linear SVM.

### Class imbalance and model support

While naive Bayes was not ideal for dealing with class imbalance without having to extend Scikit and develop more advanced methods, the other algorithms have built in support for dealing with class imbalance. The class imbalance is of paramount importance to address. The imbalance of classes in the data – there is around one Dutch user for every 100 non-Dutch users – poses a new challenge. Fortunately, both the implementations of logistic regression and the linear SVM support a class weight that adjusts weights according to the imbalance. It does so by dividing the total number of samples (documents) by the number of classes (*Dutch* or *non-Dutch*) times the frequency of the class label [22]:

(2) $$\text{weight}(y)=\frac{\#\text{samples}}{\#\text{classes}\times \#\text{occurrences}\;\text{of}\;y}.$$

#### Logistic regression

Logistic regression is a discriminative classifier, which builds a model upon the features that are most distinctive for a class [41]. The binary classifier returns well-calibrated unbiased probabilities, because it, above all, optimizes log-loss [22,42]. Logistic regression thus works with probabilities, in contrast to support vector machines. It requires the tuning of parameter *C*, which we tune by grid searching using average precision as performance measure. We find a *C* of 1 000 performs best. By default sklearn’s implementation uses *l*2-penalization. Both logistic regression and regularization come from the LIBLINEAR library [43].

#### Support vector machines

In a nutshell, the goal of an SVM is to find a separating hyperplane that is optimal, maximizing the margin of the training data. As Joachims rightly points out, SVMs are ‘universal learners’ that can learn independent of the dimensionality of the feature space [20]. Text data has many properties, such as the ones listed above, with which SVMs deal very well. Not only theoretically, but also in practice Joachims shows SVMs turn out to show good performance on text categorization tasks. Additionally, just like with logistic regression, our linear SVM only requires us to tune parameter *C*, the penalty parameter of the error term. We find *C* = 0.5 produces the highest average precision during grid search.

## Evaluation

### Cross validation

**Figure 1.**
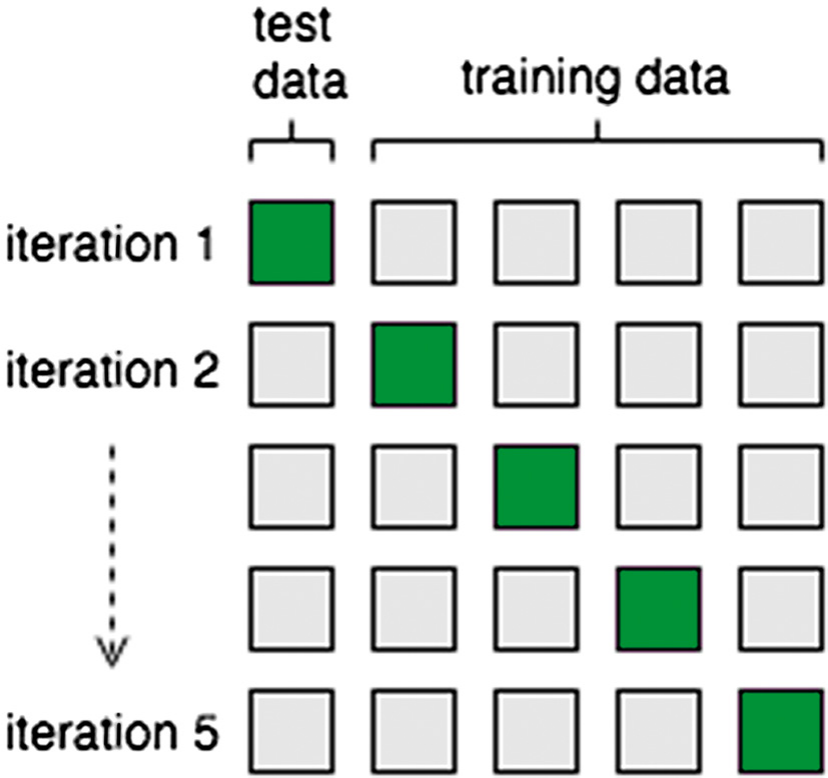
Simple five-fold cross validation.

To test the models, we use fivefold cross-validation with stratification. We stratify because stratification generally performs better than regular cross-validation, both in terms of bias and variance, according to Kohavi [44]. We apply this technique by dividing our data set in five subsets using one of the subsets as test set, the rest as training set. We repeat this process five times until every subset has been assigned test set. Because we use stratification, we take into account the balance of the classes in our division of data such that it correctly reflects the entire data set. That is, every subset has roughly the same percentage of each class as the original data set. The general cross-validation is clarified in Figure 1.

### Performance metrics

Usually, the performance of a classifier is measured by accuracy. However, in this case, the accuracy would quickly approach 99%, would a classifier always label a comment non-Dutch. So, a challenge is to find an appropriate performance metric for evaluating our model.

Fortunately, there are many ways of testing a binary classifier on how well it performs when dealing with imbalanced classes. The receiver operating characteristic curve (ROC curve), and its area under the curve (AUC) is such a way [45]. The graphical curve plots the true positive rate (TPR) against the false positive rate (FPR) at various thresholds. The AUC then tells us something about how well the algorithm does; the higher the better it does as predicting the class label. It is equal to the probability that the classifier will rank a randomly chosen Dutch instance higher than a randomly chosen non-Dutch instance [46]. However, it turns out the ROC AUC does not seem to work well for problems with many more negatives (non-Dutch) than positives (Dutch) [6]. An alternative that does not take into account the false negatives (FN) is the precision recall curve (PR curve) and its area under the curve. This metric compares FP with TP, not TN and presents the tradeoff between precision and recall. For that reason, we have chosen the PR curve and its AUC, not the ROC curve. We especially use the area under the precision recall curve, which is also known as the average precision score and describes the precision recall curve:

(3) $$\text{Precision}=\frac{\text{TP}}{\text{TP}+\text{FP}}.$$

(4) $$\text{Recall}=\frac{\text{TP}}{\text{TP}+\text{FN}}.$$

Another useful performance metric that can be used to judge the classifier on includes the *f*_1_ score which is defined as the harmonic mean of precision and recall. In our particular case, however, we would prefer to let our model find Dutch users with a high probability, then find more Dutch users but also classify many non-Dutch users as Dutch. In other words, we are willing to trade some recall for more precision. We consider precision more important than recall; we rather have false negatives than false positives. Still, with the *f*_1_ score we can keep an eye on recall as well:

(5) $$f_{1}=2\frac{\text{Precision}\times \text{Recall}}{\text{Precision}+\text{Recall}}.$$

The latter performance metrics (precision, recall, *f*_1_ score) are set-based measures, calculated using sets of comments that are unordered. On the contrary, precision recall curves are about looking for a balance between precision and recall. It works with thresholds that allow us to trade precision for recall. The performance at a range of thresholds can be visualized by plotting a precision recall curve. Its area under the curve (average precision score) then provides an excellent performance metric to compare models with.

## Experiments

The following experiments compare the performance of logistic regression with a linear support vector machine. As has been mentioned before, training is performed with Python’s Scikit-learn library on the dataset that we have obtained from Reddit. Our final dataset is significantly skewed and consists of 91 539 users and comment bases of which 852 are determined Dutch users, 90 687 non-Dutch users. Class imbalance is 1:100. We are using the parameter settings as described in Section *Related work*. The task here is to classify a comment set from a particular user as either *Dutch* (1) or *non-Dutch* (0), thus presenting a binary classification task.

## Results

The results are presented in terms of the before-mentioned *f*_1_ score, precision score, and average precision score. Moreover, stratified five-fold cross validation is performed using no feature selection, chi-squared feature selection as well as term frequency feature selection.

### Important features

First of all, both classifiers keep track of the importance of features with the respective model coefficients. If we sort these features on their importance, we obtain an interesting insight into the different word usage of Dutch and non-Dutch authors. Some of the most important features (the features with the highest coefficients) after running logistic regression – the SVM shows similar results – include terms that clearly increase the chances that a user is Dutch. These include “the Netherlands”, “dutch”, “nl”, “Holland”, etc. and some Dutch words that have slipt through the language detector as was expected. The second most important features are of a more interesting category: they are spelling errors. The model seems to have found out Dutch users make certain spelling errors that might identify them as being Dutch. These include the spelling mistakes “eachother” and “ofcourse” or “offcourse” which in fact both are to be written as two words “each other” and “of course”. “trough” is another typo or spelling error the model marks as being important as well as “focussed” which should be “focused”. Clearly, we obtain limited insight into sentence structure of Dutch writers, because we only used a unigram representation of the collected texts.

Table 1 shows the results on our Reddit dataset. As has been mentioned, three measures of performance have been used: *f*_1_, precision and average precision score.

**Table 1.** Results of experiments with two models, logistic regression and a linear support vector machine (SVM) with different feature selection methods.

Measures of performance	Logistic regression				Linear SVM			
	None	Chi2	Top 10k	Top 100k	None	Chi2	Top 10k	Top 100k
*f*_1_	0.772	0.772	0.736	0.772	0.750	0.758	0.729	0.754
Precision	0.827	0.840	0.741	0.846	0.797	0.803	0.690	0.808
Average precision	0.829	0.829	0.785	0.833	0.813	0.819	0.780	0.818

**Figure 2.**
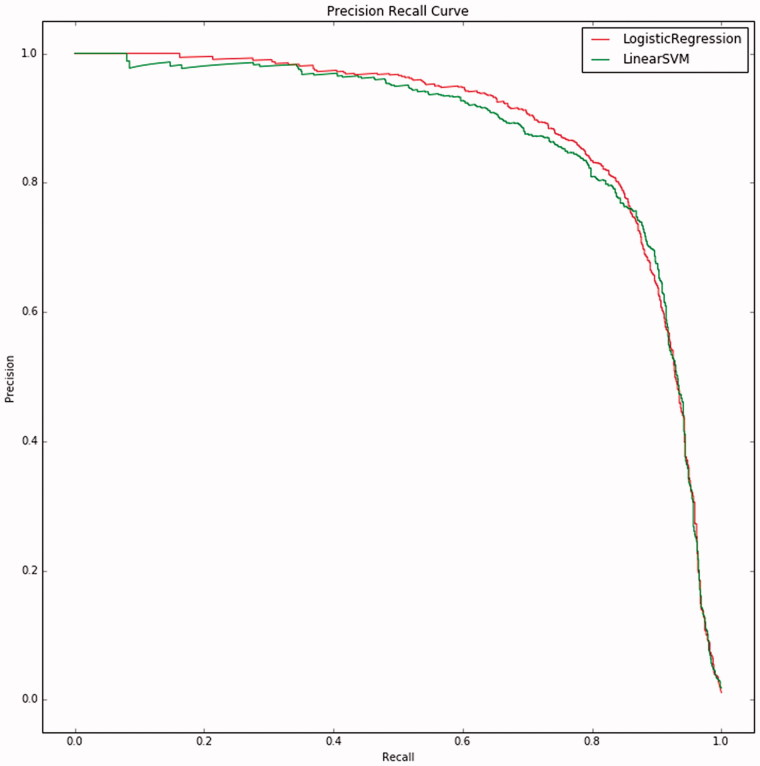
Precision/recall curves of the linear support vector machine and logistic regression. The curves have been constructed by computing precision and recall over the collected classifier outputs on the left-out parts in the cross-validation loops. The area under the precision/recall curves equals the average precision of the respective classifier.

For logistic regression, we find that *f*_1_ scores are similar across the choices of feature selection methods (0.750). Of the four test settings, logistic regression with maximum features set to 100 000 gives the best performance. To be fair, the difference of using this feature selection method or chi-squared feature selection is only slight. The precision recall curves of both models are shown in Figure 2. The figure shows that for 20% recall the precision is still almost 100% using the logistic regression models. This means that most of the documents/users that the model ranked at the top are correctly recognized as Dutch natives.

Overall, we note that logistic regression considering only the top 100 000 features scores best on every performance metric, although the difference are small.

Results in Table 1 show that logistic regression has the best results. We are not only interested in what model performs best but also how we would use the model to make sensible recommendations to intelligence agencies. To put that differently, we might not only be interested in predicting a class label, we are also interested in some sort of value that expresses a confidence on that prediction. Some models are better at giving estimates of class probabilities than others. Some even lack support for any kind of probability prediction. Fortunately, logistic regression is an algorithm that has the advantage of returning probabilities, because it directly optimizes log loss [47].

With our logistic regression model, we can advise intelligence agencies in a couple of ways, while not overlooking some important points. One option is to simply let the model classify the user that intelligence agencies provide us with. We would scrape as much comments as possible from that user and feed it to our model. Another option is to recommend based on the probabilities the logistic regression model comes up with. They both provide a tool to intelligence agencies and at the same time uncover some hidden clues that we sought after in the form of spelling mistakes.

## Conclusion

This study has posed a practical case of text classification: can we distinguish Dutch users from non-Dutch users on a typical English online forum? We have shown that with the help of popular machine learning algorithms such as logistic regression and support vector machines, a differentiation – without using any metadata – can be made between Dutch and non-Dutch users that post comments on an Internet forum. Despite the difficulty of data collection, We experimented with two models performing well on the dataset we acquired from Reddit, with *L*2 regularized logistic regression performing best. Interestingly, the models found some clues we were looking for. It found that important features included obvious Dutch words, but also common spelling errors.

Clearly, Reddit, although quite representative of an Internet forum, might not necessarily represent a forum where criminals post comments. Despite using feature selection, it is hard to determine how well the model applies to other forums. Besides, not all forums offer an easy way of gathering information. It is unlikely that many forums offer a similar API that Reddit offers. This can, however, be partially overcome by simply scraping the website with a variety of tools widely available [48]. What is hard to overcome is the fact that some forums allow users to post anonymously. Our model requires a set of comments, because it needs longer comments in order to be able to make a classification. Short comments are much harder to recognize with the proposed method. After all, we cannot gather more comments from the same user without them having some sort of name or id.

Although the proposed method has been described to detect Dutch natives on English formus, it can straightforwardly be transformed for detecting other non-English natives. Accordingly, the method can be said to be a generally applicable method.

Future research should be directed towards finding out whether the learned models can be applied to other forums with similar performance. Finally, the method can possibly be improved by for instance enabling bigrams and trigrams as text representation.
